# Supplementary material for: Antigenic Essence: Upgrade of Cellular Cancer Vaccines
Source: Cancers (Basel). 2021 Feb 12;13(4):774. doi: 10.3390/cancers13040774 (PMC7917603; doi:10.3390/cancers13040774)
Supplement: Supplementary file 1 [file cancers-13-00774-s001.pdf]

Supplementary Materials

Table S1. Summary comparison of neoantigen vaccines with antigenic essence vaccines.

**Neoantigen vaccines**	**Antigenic essence vaccines**
**Base technology**	
Genomic	Proteomic
**Fundamentals**	
Mutations in tumors are a source of specific antigens that are targeted by vaccination.	The high similarity between vaccine composition and profile of surface antigens of cancer (or cancer vasculature) cells.
**Fundamental limitations**	
Only a limited number of tumors has enough mutations to apply this technology (e.g., lung cancer and melanoma).<br>There is no direct connection between a mutation in the tumor genome and the corresponding antigen presence in the tumor. Genes may not be expressed or be expressed only weakly. To confirm that the mutated gene is expressed, a proteome analysis should be done at the stage of neoantigen selection (that is seldomly done in practice).<br>Tumor sample is required.<br>The large diversity of mutations in tumor cells means that only a personalized approach is possible, not a general one.<br>Vaccine is prepared based on genomic data of the tumor removed from the body. The vaccine is intended to prevent metastatic (repeat and secondary) tumors, which are highly likely to have their own mutations.<br>Tumor cells are genetically heterogeneous. Since the neoantigen vaccine is prepared only against a subset of tumor cells, this approach is fundamentally unable to treat a whole tumor. The remaining tumor cells, with additional mutations, will require the creation of a new vaccine (again against only some of the cells).	The cell surface profile of tumor cells changes significantly when exposed to drugs. Identification with or "matching" of the essence composition to these cell surface profiles is at the center of the vaccine design. In cases of tumor relapse, this can lead to the escape of tumor cells from the immune response induced by essence. (However, this limitation is not applicable for SANTAVAC final product that targets the tumor vasculature.)<br>SANTAVAC antiangiogenic vaccines are not suitable for blood cancers (~20% of cancer cases). Some tumors may be resistant to antiangiogenic vaccination. It is expected that some methods of tumor resistance will not work, such as splitting angiogenesis and vascular co-option, while others, such as vascular mimicry, may lead to some degree of resistance.
**Type of vaccines**	
Only personalized (technology restriction).	Any type. Antigenic essences can be autologous (obtained from the patient) as well as allogeneic.
**Type of vaccination**	
As adjuvant therapy (tumor sample is required; tumor sample usually obtained during surgical treatment of tumor).	As adjuvant therapy; as neoadjuvant therapy; as preventive vaccination.
**Production method**	
A biological sample of the tumor (paraffin section obtained for histological analysis) is used for genome sequencing (NGS of the whole exome).<br>Optional (but highly recommended) transcriptome and proteome analysis.<br>Bioinformatic treatment of genome data to predict neoantigens (search for candidates, modeling of immunogenicity and interaction with major histocompatibility complex, or MHC).<br>Chemical synthesis of several candidates (3-4 peptides).	Cell culture routines to propagate a primary culture of human microvasculature endothelial cells and produce antigens.<br>Vaccine production routines using obtained antigens.

Testing neoantigen candidates (capacity to be presented by MHC; presence in the tumor; possibility to induce an immune response).<br>Vaccine production routines using obtained neoantigens.	
**Bioinformatic treatment of data**	
Very complex (time-consuming and required for each individual patient).	Not required (only mass spectrum analysis is required for quality control of the manufactured product).
**Number of antigens used for vaccination**	
Several (3-4 peptides)	Several hundred
**Product nature**	
Chemical (neoantigens are chemically synthesized peptides; strict purification and characterization standards are required).	Natural peptides
**Universality of antigens**	
No<br>(neoantigens are strictly specific for the particular tumor of the particular patient)	Yes<br>(for antiangiogenic SANTAVAC products)
**Relation to MHC**	
Neoantigens are designed to take into account MHC restrictions	SANTAVAC compositions are designed to take into account MHC restrictions
**Relation to checkpoint inhibitors**	
Applicable	Applicable
**Targets**	
Only tumor cells with a high degree of mutation (such as lung cancer and melanoma). Tumors with a low level of mutations, such as gliomas, do not work.	Any cell type: cancer cells; endothelial cells (relate to final products); fibroblasts; etc.
**Indications**	
Only tumors with a high degree of mutations (such as lung cancer and melanoma).	Any solid tumor (~80% of cancer cases)
**Market size**	
Limited by the complexity of production, high cost of the vaccine production, restriction in the type of therapy and indications, strong fundamental limitation in efficacy.	Very high due to universality around cancer types, a wide range of production costs (from low to expensive), various available types of therapy, suitability for preventive purposes, and possibility of mass production.
**Attractiveness for big pharma**	
From Low to Average (already well-known and tested on human technology).	From Average (not yet well-known technology; preclinical stage) to High.

Conclusions

Antigenic essence (SANTAVAC) vaccines demonstrate many features of mainstream technology but free of some fundamental restrictions, with expected high efficacy, high market coverage, and capacity for bulk production by big pharma.

Neoantigen vaccines are very complex to produce, personalized-only (not only from the points of efficacy, but rather as limitation of the approach), very expensive, very time-consuming to produce, and intended to treat a limited number of tumors. While the fundamentals of this type of vaccine are attractive, limitations of the technology suggest that the overall efficacy of such vaccines is likely to remain elusive.
